# A Multifunctional Network with Uncertainty Estimation and Attention-Based Knowledge Distillation to Address Practical Challenges in Respiration Rate Estimation

**DOI:** 10.3390/s23031599

**Published:** 2023-02-01

**Authors:** Kapil Singh Rathore, Sricharan Vijayarangan, Preejith SP, Mohanasankar Sivaprakasam

**Affiliations:** 1 Indian Institute of Technology Madras, Chennai 6000001, India; 2 Healthcare Technology Innovation Center, Chennai 6000001, India

**Keywords:** deep learning, respiration rate, wearable technology, multifunctional network, attention mechanism, uncertainty estimation, model compactness

## Abstract

Respiration rate is a vital parameter to indicate good health, wellbeing, and performance. As the estimation through classical measurement modes are limited only to rest or during slow movements, respiration rate is commonly estimated through physiological signals such as electrocardiogram and photoplethysmography due to the unobtrusive nature of wearable devices. Deep learning methodologies have gained much traction in the recent past to enhance accuracy during activities involving a lot of movement. However, these methods pose challenges, including model interpretability, uncertainty estimation in the context of respiration rate estimation, and model compactness in terms of deployment in wearable platforms. In this direction, we propose a multifunctional framework, which includes the combination of an attention mechanism, an uncertainty estimation functionality, and a knowledge distillation framework. We evaluated the performance of our framework on two datasets containing ambulatory movement. The attention mechanism visually and quantitatively improved instantaneous respiration rate estimation. Using Monte Carlo dropouts to embed the network with inferential uncertainty estimation resulted in the rejection of 3.7% of windows with high uncertainty, which consequently resulted in an overall reduction of 7.99% in the mean absolute error. The attention-aware knowledge distillation mechanism reduced the model’s parameter count and inference time by 49.5% and 38.09%, respectively, without any increase in error rates. Through experimentation, ablation, and visualization, we demonstrated the efficacy of the proposed framework in addressing practical challenges, thus taking a step towards deployment in wearable edge devices.

## 1. Introduction

Respiration rate (RR) has long been one of the most promising parameters to assess the exertion of the body and its response to load variations during physical activities [1,2]. RR is traditionally measured using classical methods such as impedance pneumography and capnography. However, despite high accuracy, these methods require an obtrusive setup, making them burdensome for computation during daily life activities. Hence, there is a requirement for an unobtrusive setup to estimate RR during such activities. It has been demonstrated that RR can be estimated with reasonable accuracy using physiological signals such as electrocardiogram (ECG), photoplethysmography (PPG), accelerometer, etc. [3,4]. However, all these techniques have certain limitations, such as source signal becoming corrupted due to noises or variation in individual respiration modulation between patients, etc. [5].

The inherent limitation of each technique can be suppressed using fusion [3]. In order to implement fusion algorithms, research studies such as [6,7,8] utilized the representative power of machine learning (ML)/deep learning (DL) algorithms for accurate respiration rate estimation.

The study proposed by [9] tested a variety of DL models to prove the efficacy of these algorithms for RR estimation. Furthermore, an ML-based smart fusion (SF) technique to fuse the individual RR estimates from the PPG and ECG based on their respiratory quality index was proposed by [8]. A similar study was proposed by [10] using neural networks. A linear regression (LREG) algorithm was proposed by [11] to estimate RR from ECG and accelerometer. A convolutional neural network (CNN)-based architecture to estimate the RR from PPG was proposed by [12]. However, the problem formulation used in these studies allowed the estimation for a fixed window size only. It did not compute individual respiration peaks, which was useful for fetching the instantaneous RR. RespNet (RN) [13] solved this limitation by estimating the respiration signal from PPG using a Unet-based architecture, which allowed the detection of individual breathing peaks. However, the RN required significant parameters and thereby required more extensive storage.

Moreover, all these studies were performed on clinical data, and their efficacy on ambulatory data was not studied. Rathore et al. [14] proposed a multitasking model (MRNet) to overcome these issues. The MRNet required the respiration signals derived from ECG and accelerometer as input and gave fused respiration signal and average RR simultaneously as outputs. The network showed reasonable accuracy on the ambulatory dataset while consuming fewer parameters than RN. However, the model took a considerable amount of parameters, and it lacked the capability to work in the case of highly dynamic ambulatory activities. Additionally, to make a DL model practically realizable, technical aspects such as attention-based visualization mechanism and uncertainty estimation need to be embedded into a single framework. In order to deploy the model on an edge device, it is also necessary to develop a DL model consuming optimal storage and runtime while maintaining a similar level of prediction accuracy [3].

A typical DL model works as a black box where internal functioning and interpretation is unavailable. Additionally, a DL model processes the entire input irrespective of noise and other irrelevant information. Hence, there is a need for a mechanism that shows the interpretability of the internal process and prunes the irrelevant features. Previous works such as [15] used input selection algorithms to consider the relevant regions of the input. However, using separate input selection algorithms before feeding to a DL model increases the overall computation overhead of the system. This drawback is solved by using attention blocks which not only remove the irrelevant regions, consuming comparatively less time, but also aid in showing the interpretability of the modeling process. The studies carried out in [16,17] demonstrated the effectiveness of using attention blocks for regression-based problem formulation. Additionally, the attention blocks can be easily embedded into any DL architecture and trained using standard backpropagation.

During ambulatory activities, it is imperative to quantify the uncertainty associated with final RR estimates [3] so that RR estimates with high uncertainty scores can be discarded. The input-based rejection methods to filter the unreliable estimates were proposed in [8,18]. While these methods can increase the accuracy of the final prediction, they account for the uncertainty that is introduced primarily due to the noisy input data. However, a DL model may draw reliable insights from the noisy input and predict an output higher in accuracy but with more uncertainty [19]. Hence, for a DL model, it is important to consider the uncertainty introduced due to both data and model prediction, namely, aleatoric and epistemic uncertainty, respectively. Previous studies such as [5] resolved this problem with the help of the Gaussian process. Taking inspiration from this work and integrating it with DL’s rich representative power, Monte Carlo (MC) dropout, a popular method used in medical imaging [20,21] based on the Gaussian process, is used to estimate uncertainty in the predictions. MC dropouts account for both aleatoric and epistemic uncertainty. The prediction with higher uncertainty can be ignored, reducing the final estimation error. The uncertainty estimation can be obtained by training the network with a standard backpropagation technique.

With the advancement in embedded devices that run DL models at inference, such as Raspberry Pi 4, NVIDIA’s Jetson nano, etc., it is important to decrease the model’s size and reduce the time required for execution [3,22]. For this purpose, we used knowledge distillation (KD) to decrease our model’s size and inference time without a significant drop in performance. Previous studies used different methods such as pruning and quantization, low-rank approximation, and transferred convolution filters to compress the model size [23]. Pruning and quantization do not improve the accuracy after parameter reduction, while low-rank approximation and sparsity-based methods are difficult to implement. Meanwhile, transferred convolution filters feasibly work only for wide networks. Of all the methods, KD works the most efficiently on deep networks to compress a large model without compromising accuracy [23]. The KD procedure involves a teacher and a student model. The student model is a smaller-sized derivative of the teacher model. The student model learns from the teacher model to compensate for its accuracy, which drops due to parameter reduction. Consequently, a small-size student model produces comparable accuracy with reference to the teacher model with optimal inference time and parameter count.

Our aim is to develop a DL framework embedded with the above discussed functionalities and demonstrate its relevance for accurate estimation of RR and respiratory signal during ambulatory activities, taking it a step closer towards deployment in wearable and edge devices. Our contributions are summarized as follows:

1. We propose an attention block embedded multitasking network (ATTMRNet), which estimates average and instantaneous RR. Crucially, we discuss and demonstrate the improvements in estimating RR brought about by the interpretation of the internal processes by the attention block.
2. We propose applying an MC-dropouts-based uncertainty estimation technique embedded in the same architecture, such that the estimates with high uncertainty can be discarded. Furthermore, we also elaborate on the trade-off between RR window rejection, error rate reduction, and the number of inference runs required.
3. We propose the application of an attention-based KD technique to optimize the inference time and parameter count, making it more viable for deployment on wearable edge devices.
4. We demonstrate the effectiveness of the entire framework, which includes the attention block, MC dropout, and KD, through extensive comparisons with previous state-of-the-art methods and baselines. We used the Incremental Running dataset (IR dataset), which was collected in-house, and the publicly available PPG-DaLiA dataset, both of which contain ambulatory activities that closely simulate real-world environments.

## 2. Related Work

### 2.1. Respiration Signal and RR Extraction

The first step mainly focuses on extraction of respiration signal from physiological signals such as ECG, PPG, and accelerometer signals. A typical algorithm traces amplitude modulation, frequency modulation, and baseline wander to extract respiration signals from ECG and PPG. These algorithms are classified as filter-based algorithms and feature-based algorithms. The filter-based algorithms use techniques such as empirical ensemble mode decomposition [24], homomorphic filtering [25], etc. The feature-based technique extracts fiducial points of an ECG, PPG waveform. These include extracting R peak amplitude and R–R-interval-based respiration signal derivation [26], and respiration signal extraction by tracing the QRS durations [27]. A detailed review of such methods is presented in [3].

An accelerometer signal is also used to extract the respiration signal. Liu et al. [28] presented a method based on principal component analysis to extract the respiration signal from a three-dimensional accelerometer. An adaptive filter-based technique was presented by [4] to extract a respiration signal from the accelerometer.

The majority of these algorithms have some inherent limitations, such as motion artifacts. We can suppress the inherent limitation of such individual methods through fusion, as described in [3].

Fusion-based algorithms involve the fusion of respiration signals from individual modalities or individual RR estimates to obtain the final estimate. The basic fusion techniques such as averaging individual respiration rate estimates are implemented in [18]. Some advanced signal processing methods such as autoregression modeling, pole magnitude criteria, etc., are also implemented in previous research studies. Jarchi et al. [29] used autoregressive modeling and fast Fourier transform to fuse the PPG and triaxial accelerometer for respiration rate monitoring of post-intensive-care patients. All these studies provided a robust estimate of the final RR for the corresponding application, but further improvement in the accuracy of final respiration estimation can be made by utilizing the representative power of ML and DL algorithms, as these algorithms have the flexibility to adapt to the datasets with high variance.

### 2.2. Machine Learning and Deep Learning Based Techniques

Birrenkott et al. [8] used ML algorithms such as random forest regression, support vector regression, etc., to create a smart fusion that fuses the RR obtained from different modulations of ECG and PPG. An ML-based approach to extract RR from headworn PPG was presented by [30]. Chan et al. [11] used linear regression to fuse the respiration signal obtained from ECG and accelerometer. Pimentel et al. [5] used Gaussian regression, a probabilistic approach of regression, to estimate RR from the PPG signal.

While the ML-based algorithms are fairly accurate, it has been demonstrated that DL algorithms perform better in terms of accuracy.

Different DL algorithms were introduced to estimate the respiration signal or RR. Kumar et al. [9] conducted a thorough study to prove the efficacy of DL algorithms for RR estimation. The study described by [10] used the neural network to fetch RR from ECG and PPG. A ConvMixer network was proposed by [31] to fetch RR from PPG. A CNN was proposed in [12] to estimate RR from the PPG signal. However, this method does not provide instantaneous breathing cycles. This problem is addressed in RN, presented in [13], which extracts the respiration signal from PPG. However, the architecture proposed in the study has a large parameter count, which increases the computation time.

Additionally, these studies provided reliable results on clinical datasets, but their efficacy on ambulatory datasets was not studied. A deep-learning-based multitasking network was presented in [14], which gives both instantaneous and average RR. When tested on the nonstationary dataset, this network performed better than previously developed works. However, the scope for improvement in the accuracy for high-intensity activities is still there. During highly dynamic ambulatory activities, the source input signal is contaminated with high noise, which affects the output. Hence, an automated modality is needed in the DL model to prune the noisy part of the input and enhance the accuracy of the final estimate. This aspect can be formulated by adding an attention block such that the model processes the input by ignoring the noisy part and highlighting the relevant features to reduce the error.

### 2.3. Attention-Based Techniques

Attention-based techniques have been used in biomedical imaging to retrieve the salient features of an input image to enhance the overall accuracy. Oktay et al. [32] presented a gated-signal-based attention block embedded into a Unet for pancreas segmentation. A spatial-attention-gate-based technique was presented by [33] to segment the abnormal tissues. An attention-guided deep regression network based on two Unet frameworks for landmark detection in cephalograms was proposed in [16]. Alzahrani et al. [17] proposed an attention-mechanism-guided regression model for acne grading. These studies proved the utility of attention blocks in pruning the irrelevant features and highlighting the salient ones.

However, for estimation during ambulatory activities, it is important to obtain predictions with a degree of confidence, such that highly uncertain predictions can be ignored. Thus, along with attention blocks, we couple the same architecture with an uncertainty estimation module to obtain a better degree of confidence in the prediction.

### 2.4. Uncertainty Estimation Techniques

Many research studies have employed uncertainty estimation, especially in biomedical images. Uncertainty estimation in RR estimation was previously introduced by [5] using Gaussian regression. In the case of DL, the concept of deep ensembles was introduced by [34]. A Bayesian deep-learning-based approach was presented in [19]. This approach formulates the Bayesian approximation by adding dropouts in the network. The application of these two approaches has been implemented in many regression tasks [20,21]. Bayesian approximation’s formation by adding dropouts is highly automated and modular and can be embedded into any DL architecture; however, it comes at the cost of a large inference time. Hence, it is imperative to add functionality to compress the deep learning model without compromising its accuracy to use the memory optimally. Hence, the concept of knowledge distillation comes into the picture.

### 2.5. Knowledge Distillation Techniques

KD is used in biomedical images for compressing a large model to optimize its storage and reduce inference time. A detailed review of KD and its application is provided in [22]. KD was used to compress the models built for medical image segmentation in [35,36,37]. An attention-transfer-based KD technique was also presented in [38], and its application for compressing the architecture of MRI reconstruction was presented in [39]. All these works showed the effectiveness of KD in model compression. Improvement in the accuracy of the student model was also reported in previous studies [22] by using KD, making it a suitable technique for model compression for our study.

## 3. Dataset Description

### 3.1. PPG-DaLiA Dataset

The PPG-DaLiA dataset was collected from 15 subjects; seven males and eight females aged between 20 and 40 years [7]. The dataset comprises physiological data from wrist and chest-worn devices. We used the ECG, accelerometer, and respiration signals from the chest-worn device for our study. All the signals were recorded at a sampling frequency of 700 Hz. The dataset also contains annotated R peak locations.

The data collection protocol included different activities such as sitting, climbing stairs, cycling, etc. Data from one subject were discarded because of improper recording due to a hardware issue [7].

### 3.2. Incremental Running Dataset

The dataset was collected in accordance with Bruce protocol [40]. The subjects were asked to run in multiple stages of speed and inclination on the treadmill till exhaustion. Since this protocol requires high-intensity running, we considered twenty-one male subjects leading an active lifestyle aged 25 ± 3 years for the study. The subjects were asked to avoid any high-intensity activity 24 h before the data collection. Initially, the subjects were asked to sit for 10 min to record their baseline heart rate. Later, the subjects were asked to walk at a slow pace for one minute as a warm-up. Further, the subjects were made to run with speed, and grade was ramped up every three minutes, as shown in Table 1.

The subjects were asked to run till they were physically exhausted. After this, the subjects were asked to walk at a slow pace for one minute as a cool-down. Finally, the subjects were asked to sit for 10 min for their heart rate to reach its base line value.

We used the Equivital EQO2 life monitor (Hidalgo, Swavesey, UK) to acquire ECG, accelerometer, and respiration signal. Previously, this device was used in many research studies such as [24,41]. The ECG and accelerometer signals were recorded at 256 Hz, while the reference respiration signal was recorded at 25.6 Hz.

## 4. Methodology

### 4.1. Respiration Signal and Respiration Rate Extraction

The ECG and accelerometer signals were divided into 32 s windows [8]. The PPG-DaLiA dataset (Section 3.1) contained R peak amplitude (RPA) and R–R interval (RRint), and, on the other hand, the same was obtained for the IR dataset by applying the neurokit tool [42]. The respiration signal was then extracted by applying the algorithm given in [26] on RPA and RRint.

The accelerometer-based respiration signal (ADR) for both datasets was derived using the adaptive filter approach given in [4]. The individual respiration signal obtained from different modalities was resampled to 4 Hz [26]. The respiration rate from the three respiration signals obtained from ECG, accelerometer, and the reference respiration signal was calculated using an advanced counting algorithm [43]. The algorithm detects the relevant breathing peaks which were used to obtain the instantaneous and average respiration rate (duration between the first and last peak of 32 s).

### 4.2. Problem Formulation

#### 4.2.1. Multitasking Functionality Problem Formulation

The base multitasking model takes the respiration signal from individual modalities as input and outputs the point estimate of respiration signal and the average respiration rate for a 32 s window size. The inputs $X=\left(x^{\left(11\right)},x^{\left(12\right)},y^{\left(11\right)},y^{\left(12\right)}\right),(x^{\left(21\right)},x^{\left(22\right)},y^{\left(21\right)},$ $y^{\left(22\right)}),\ldots ,\left(x^{\left(n1\right)},x^{\left(n2\right)},y^{\left(n1\right)},y^{\left(n2\right)}\right)$, which is ECG- and accelerometer-derived noisy respiration signal, were fed into the DL model to obtain ${\hat{y}}^{\left(n1\right)}$, the fused respiration signal waveform, and ${\hat{y}}^{\left(n2\right)}$, a point estimation of average RR, where ($x^{\left(i\right)},y^{\left(i\right)}\in R^{n}$). The encoder downsamples the input multiple times to produce the compressed featured vector $Z^{\left(i\right)}$. The decoder upsamples the bottleneck layer’s feature vectors to fetch the final respiration signal. Simultaneously, the IncResNet downsamples the same to output the average respiration rate. The mathematical representation of the model is given below:

(1) $$z^{\left(i\right)}=E_{1}\left(x^{\left(i\right)};\theta_{1}\right)$$

(2) $${\hat{y}}^{\left(i1\right)}=D_{1}\left(z^{\left(i\right)};\theta_{2}\right)$$

(3) $${\hat{y}}^{\left(i2\right)}=I_{1}\left(z^{\left(i\right)};\theta_{3}\right)$$

where $E_{1}$, $D_{1}$, and $I_{1}$ represent the encoder, decoder, and IncResNet block with dense layer having parameters $\theta_{1}$, $\theta_{2}$, and $\theta_{3}$, respectively. The optimization of the weights and biases in the architecture was performed by minimizing the smooth $L_{1}$ loss between ${\hat{y}}^{\left(ni\right)}$ and $y^{\left(ni\right)}$.

The loss function L(X) is defined as:

(4) $$L_{1}\left(X\right)=\sum_{i=1}^{m}L_{sl1}\left(y^{\left(i1\right)}-{\hat{y}}^{\left(i1\right)}\right)$$

(5) $$L_{2}\left(X\right)=\sum_{i=1}^{m}L_{sl1}\left(y^{\left(i2\right)}-{\hat{y}}^{\left(i2\right)}\right)$$

Equation (4) minimizes the loss function between the predicted respiration signal obtained at the decoder and ground truth respiration signal, whereas Equation (5) minimizes the loss function between the predicted average RR obtained at IncResNet block and ground truth.

Since both average and instantaneous RR are obtained simultaneously, the network minimizes the overall loss function, which can be written as:

(6) $$L_{total}\left(X\right)=L_{1}\left(X\right)+L_{2}\left(X\right)$$

where $L_{sl1}\left(x_{n},y_{n}\right)$ is the smooth *L*1 loss defined below:

(7) $$L_{sl1}\left(x_{n},y_{n}\right)=\left\{\begin{array}{cc} 0.5{\left(x_{n}-y_{n}\right)}^{2}, & if|x_{n}-y_{n}|\;<1\\ |x_{n}-y_{n}|-0.5, & \mathrm{otherwise}, \end{array}\right.$$

The smooth *L*1 loss works similar to an absolute error function (*L*1-loss) when the absolute value of the argument is greater than one. It works as a squared error function (*L*2-loss) when the absolute value of the argument is less than one. This robust property of smooth *L*1 loss makes it an appropriate choice for the proposed model.

#### 4.2.2. Problem Formulation for Attention Blocks

**Figure 1:**
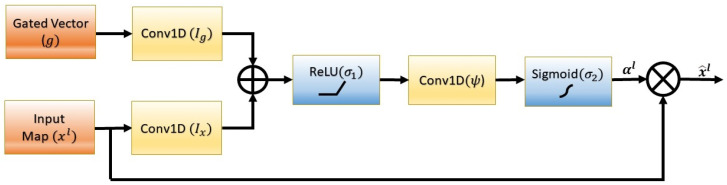
A series of transformations to process the input features and the gating vector to generate the attention coefficients. The input features are scaled by attention coefficients so that the relevant part of the inputs is highlighted and the irrelevant part is suppressed.

The attention blocks highlights the features relevant to the specific tasks using attention coefficients $\alpha_{i}\in \left[0,1\right]$. The block diagram of a unit attention block is shown in Figure 1.

As shown in Figure 1, for a layer *l*, the output of the attention block is the element-wise scalarmultiplication of the input feature map and the attention coefficients as given below:

(8) $${{\hat{x}}_{i}}^{l}={x_{i}}^{l}.{\alpha_{i}}^{l}$$

Attention coefficients are generated by the input feature map (${x_{i}}^{l}\in R$) and a gating vector ($g_{i}\in R$). The gating vector carries the activation and contextual information obtained at the lower level [32], helping to select the relevant features for a specific task. The attention coefficients are calculated for each feature map vector. The overall attention coefficient generation is a two-step sequential process.

In the first step, the input feature maps and gated vector are passed through a linear transformation to process the information layer by layer. The linear transformation for our problem formulation is performed with the help of 1D convolution layers. Further conditioning of the information obtained from the large receptive field is carried out by applying the ReLU activation ($\sigma_{1}$) and another 1D convolution layer (ψ). Mathematically, this step can be written as given below:

(9) $${O^{l}}_{att}=\psi^{T}\left(\sigma_{1}\left({I_{x}}^{T}{x_{i}}^{l}+{I_{g}}^{T}g_{i}+b_{g}\right)\right)+b_{\psi }$$

where ${x_{i}}^{l}\in R$ is input feature map, $g_{i}\in R$ is gating vector, $I_{x}\in R$ and $I_{g}\in R$ are 1D convolutional linear transforms, $b_{g}\in R$ and $b_{\psi }\in R$ are the bias terms, $\sigma_{1}$ represents ReLU activation, and $\psi^{T}$ is the second 1D convolution applied after ReLU activation, as shown in Figure 1.

In the second step, the sigmoid activation ($\sigma_{2}$) is applied to the intermediate output ${O^{l}}_{att}$ obtained in the first step. The attention coefficients for a layer *l* can be obtained as shown below:

(10) $${\alpha_{i}}^{l}=\sigma_{2}\left({O^{l}}_{att}\right)$$

where,

(11) $$\sigma_{2}\left(x_{i}\right)=\frac{1}{1+e^{-x_{i}}}$$

The output of the sigmoid activation function is the normalized final attention coefficients; therefore, these attention coefficients can be treated as weights. These weights are generated by the gating signal obtained at the coarse level. Therefore, the more relevant the features, the higher the value of the attention coefficient, and vice versa. Consequently, the scalar multiplication of attention coefficients with the feature maps, as given by Equation (8), suppress the irrelevant region of the feature map and point out the relevant features to enhance the output accuracy.

#### 4.2.3. Uncertainty Estimation Using MC Dropouts

Consider a supervised DL model problem formulation with dataset D={X,Y}, where $X=\{x_{1},x_{2},\ldots ,x_{N}\}$ and $Y=\{y_{1},y_{2},\ldots ,y_{N}\}$. A typical DL problem formulation establishes a functional mapping *f* between *X* and *Y* parameterized by a set of weights *W*, by minimizing the sum of square error loss function. This optimization problem can be written as:

(12) $$\min_{W}J\left(W\right);\;\;J\left(W\right)=\frac{1}{N}\sum_{i=1}^{N}L_{i}\left(W\right)$$

where $L_{i}\left(W\right)$ is the sum of square error loss function, described as:

(13) $$L_{i}\left(W\right)=\frac{1}{2}\left|\right|y_{i}-f\left(x_{i};W\right){\left|\right|}^{2}$$

With the deterministic problem formulation shown in Equation (12), a DL model trains to learn the mean answer but it does not formulate the uncertainty in data while making the prediction.

This problem can be solved by modeling the distribution over weights *W* using Bayesian neural networks, which allows the model to learn the mean and variance by maximizing the likelihood estimation of the model. Consider the same dataset *D*. We would like to predict the output $y^{*}$ for an input vector $x^{*}$; the predictive posterior distribution of $y^{*}$ can be written as:

(14) $$P\left(y^{*}|x^{*},D\right)=\int P\left(y^{*}|x^{*},W\right)P\left(W|D\right)dW$$

where $P(y^{*}|x^{*},D)$ is the model’s likelihood and P(W|D) is posterior over the weights.

The predictive posterior distribution includes both mean and variance, yielding the uncertainty estimation. The integral in Equation (14) shows predictive distribution, and it is computationally expensive for most networks. However, the posterior distribution can be solved by approximating the variational inference $q_{\theta }\left(W\right)$, parameterized by θ. Now Equation (14) can be rewritten as:

(15) $$q_{\theta }^{*}\left(D\right)=\int P\left(y^{*}|x^{*},W\right)q_{\theta }\left(W\right)\approx P\left(y^{*}|x^{*},D\right)$$

The variational inference $q_{\theta }\left(W\right)$ is obtained by fitting the Gaussian process such that it is close to P(W|D), and this is achieved by minimizing the Kullback–Leibler (KL) divergence between the two distributions:

(16) $$KL\left(q_{\theta }\left(W\right)|P\left(W|D\right)\right)=\int q_{\theta }\left(W\right)\log\left(\frac{q_{\theta }\left(W\right)}{P(W|D)}\right)dW$$

The implementation of variational inference depends on the type of variational distribution; therefore, solving Equation (16) might create a significant bias for expressive distribution. The application of MC dropouts [19] can be used effectively to solve variational inference in Bayesian neural networks. We can define $q_{\theta }\left(W_{i}\right)$ as the approximation distribution for weights for each layer *i*. It can also be taken as the distribution over matrices, whose columns are randomly set to zero by applying the Bernoulli distribution, expressed as:

(17) $$W_{i}=M_{i}.diag\left({\left[Z_{i,j}\right]}_{j=1}^{K_{i}}\right)$$

where $Z_{i,j}$∼$Bernoulli(p_{i})$ for $i=1,\ldots ,L,j=1,\ldots ,K_{i-1}$, each $Z_{i,j}$ is a Bernoulli random variable deciding whether a connected input should be dropped or not with probability $p_{i}$. Taking the $q_{\theta }\left(W\right)$ as the approximate distribution over the weights and utilizing the equivalency of variational inference using MC dropouts with the Gaussian process fitted to the neural network with the model precision τ and length scale *l* [19], the approximate predictive posterior defined in Equation (14) along with the mean and the variance can be written as:

(18) $$P\left(y*|x*,D\right)=N(y^{*};f^{W}\left(x^{*}\right),\tau^{-1}I)$$

(19) $$E_{q_{\theta }\left(y^{*}|x^{*}\right)}\approx \frac{1}{T}\sum_{t=1}^{T}f^{W}\left(x^{*}\right)$$

(20) $$Var_{q_{\theta }\left(y^{*}|x^{*}\right)}=\tau^{-1}I_{D}+\frac{1}{T}\sum_{t=1}^{T}{\left(f^{W}\left(x^{*}\right)\right)}^{T}f^{W}\left(x^{*}\right)-{\left(E_{q_{\theta }\left(y^{*}|x^{*}\right)}\left(y*\right)\right)}^{T}\left(E_{q_{\theta }\left(y^{*}|x^{*}\right)}\left(y*\right)\right)$$

The mean and variance in Equations (19) and (20), respectively, are predictive parameters of the model using MC dropouts. This mean and variance are obtained by running the model’s *T* stochastic forward passes during test time and then averaging the results.

#### 4.2.4. Problem Formulation for Knowledge Distillation

Knowledge distillation includes transferring the information obtained from the feature maps acquired from the teacher model to the student model, such that the student model shows comparable accuracy concerning the teacher model with a lower parameter count and consuming less time. The teacher model $M(t;\phi_{t})$ is the original bigger size model, and the student model $M(st;\phi_{st})$ is the smaller-sized derivative of the teacher model. The functioning of KD is explained as follows:

Firstly, the teacher model $M(t;\phi_{t})$ is trained with respect to the ground truth while minimizing the smooth *L*1 loss ($L_{sl1}\left(x_{gt},x_{t}\right)$) given in Equation (7), where $x_{gt}$ are the ground truth values and $x_{t}$ are the teacher model’s prediction.

In the next step, the feature-based distillation of the acquired attention maps obtained from the pretrained teacher model $M(t;\phi_{t})$ to the untrained student model $M(st;\phi_{st})$ is performed. The attention map of the features is given as $F_{sum}\left(A\right)=\sum_{i=1}^{C}{\left|A_{i}\right|}^{2}$. To obtain the effective feature distillation, we opted for the following attention transfer loss [38]:

(21) $$L_{AT}=\sum_{j\in I}\left|\right|\frac{Q_{st}^{j}}{\left|\right|Q_{st}^{j}{\left|\right|}_{2}}-\frac{Q_{t}^{j}}{\left|\right|Q_{t}^{j}{\left|\right|}_{2}}{\left|\right|}_{2}$$

where $Q_{st}^{j}=vec\left(F_{sum}\left({A_{st}}^{j}\right)\right)$ and $Q_{t}^{j}=vec\left(F_{sum}\left({A_{t}}^{j}\right)\right)$ represents the *j*-th pair student and teacher attention maps in the vectorized form, and *I* denotes the set of teacher and student model’s convolution layers selected for the attention transfer. In our framework, *j* distills the attention maps from all the layers by iterating through the feature from the whole architecture. The attention-transfer-based feature distillation step to train the student network is required to match the attention maps of the student model with the teacher model.

In the next step, the pretrained student model $M(st;\phi_{st})$ optimizes the loss function described below:

(22) $${L_{total}}^{st}=\alpha L_{sl1}\left(x_{gt},x_{st}\right)+\left(1-\alpha \right)L_{sl1}\left(x_{t},x_{st}\right)$$

where $L_{sl1}$ represents the smooth *L*1 loss as given in Equation (7); $x_{gt}$ represents the ground truth values, $x_{t}$ represents the teacher model’s prediction, and $x_{st}$ represents the student model’s prediction. The second term in Equation (22) is an imitation loss term [39]; it acts as the regularizer to the loss between the ground truth and student’s prediction (first term of Equation (22)) with the factor α. The regularization by imitation loss improves the performance of the student model with optimized parameter count and consumes the optimal time.

### 4.3. Model Architecture

**Figure 2:**
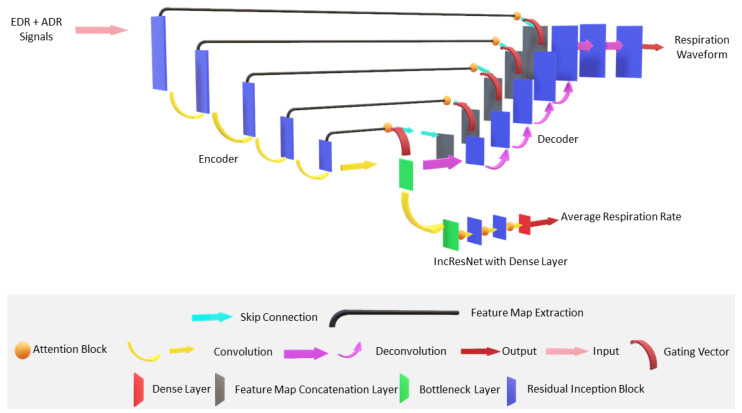
Proposed model architecture. The architecture is divided into three parts, namely, encoder, decoder, and IncResNet with dense layer. Other functionalities are embedded in the same architecture.

The base architecture for ATTMRNet is inspired by the architecture developed in our previous work [14]. The model architecture consists of an encoder, a decoder, and an IncResNet block with a dense layer for the multitasking functionality. The base architecture contains five encoder–decoder levels ($n_{ed}=5$). The basic diagram of the model is shown in Figure 2.

The attention blocks are embedded into the same architecture to select the relevant features and introduce interpretability, as shown in Figure 2.

The MC dropout is placed in the model to estimate the uncertainty associated with the final prediction. In order to reduce the parameter count and inference time, we derived the smaller-sized student model considering the model shown in Figure 2 as the teacher model. The distillation of feature maps from the teacher to student model was performed by KD as described in Section 4.2.4. All the hyperparameters of the architecture were fixed empirically. The details of the architecture are described as follows:

#### 4.3.1. The Encoder Block

The encoder was designed with multiple 1D convolution layers to downsample the input respiration signal. Each 1D convolution layer of the encoder was appended with a batch normalization layer, an ReLU activation layer having a slope of 0.2, and an Inception-Res block. The number of filters in the 1D convolution layer was initially set to 32 and then increased by a factor of 2 for each subsequent layer until the bottleneck layer, with a final filter size of 1024. While downsampling, the kernel size, and stride for all the layers were fixed to 3 and 2, respectively.

#### 4.3.2. The Decoder Block

In order to output the respiration signal, the features collected at the bottleneck layer were upsampled by the decoder block. For upsampling, the multiple 1D transposed convolutions were used. Batch normalization, Leaky ReLU activation with the slope of 0.2, and Inception-Res block were appended to the 1D convolution transposed layer. The filter size was initialized to 512 and reduced by a factor of 2 for every subsequent block with a kernel size of three and stride 2.

#### 4.3.3. IncResNet Block

This block was designed to downsample the bottleneck features to output the average respiration rate. The design specification of this block is similar to that of the encoder. Here, the initial filter size of the 1D convolution layer was set to 128. For the next two layers, the filters were reduced by a factor of two, and later by 16, with kernel of size 4 and stride 2. Finally, a unit-dense layer was added at the end.

#### 4.3.4. Inception-Res Block

This block, which contains four 1D convolution layers, was added to diminish the vanishing gradient problem. Batch normalization and Leaky ReLU with a slope of 0.2 were applied after each convolution layer. We used the same filter size, which is determined by taking one-fourth of the number of output channels for all the four convolution layers. The kernel size was set to 15, 17, 19, and 21 for the four 1D convolution layers.

#### 4.3.5. Design and Placement of Attention Block

The design of the attention block given in Figure 1 consists of a 1D convolution operation of the input feature map and gating vector. The sum of these two intermediate outputs was passed through ReLU activation, followed by another 1D convolution, followed by sigmoid activation to obtain the attention coefficients. The filter size of the 1D convolution layer was fixed to 1 with kernel size and stride of length 1. In order to obtain the respiration signal from the relevant features, the attention block was placed such that the gating vector is taken as input from the lower layer and the feature map from the encoder as shown in Figure 2. During feature map concatenation, using skip connection, the pruned features obtained from the attention blocks are passed to the decoder. The self-attention functionality of the attention blocks was utilized [32], and the placement was carried out in a cascaded manner in the IncResNet block to obtain the average RR, as shown in Figure 2.

#### 4.3.6. Placement of MC Dropouts

The MC dropouts were added into the encoder, decoder, and IncResNet block of the proposed model as per the convention described in [44]. The dropouts were placed after the Leaky ReLU activation layer in all the three blocks, with the dropout probability fixed as 0.1. The same dropout probability is used in various convolution neural network-based architecture [45]. They are then activated during the inference to obtain the uncertainty estimation.

#### 4.3.7. Student Model Design for KD

The smaller-sized student model was derived from the main teacher model given in Figure 2 by randomly removing residual inception blocks from the encoder, decoder, and IncResNet blocks. A level of three for the encoder–decoder ($n_{ed}=3$) was considered for the design. This $n_{ed}=3$ was selected as per experimentation described in Section 5.4. For the student model, the initial filter size of the 1D convolution layer of the encoder was fixed to 32 and increased by a factor of two till it reached 128 with a kernel size of three and a stride of 1. The number of filters in the bottleneck layer was fixed to 1024, the same as that of the teacher model. The filter size in the decoder, having an initial filter size of 128, was then reduced by a factor of two till it reached 32 and then reduced by 8 and 4 to obtain the final respiration signal output. The kernel size for the remaining 1D convolution transposed layer of the decoder was fixed to 3, and the stride was taken as 1.

For the IncResNet block, the initial number of filters was fixed at 128 with a kernel size of four and a stride of four. Firstly, the filter size was reduced by a factor of 4 with kernel size four and stride 2. Finally, the number of filters was reduced by a factor of 8, followed by a unit dense layer. The 1D convolution layer for the encoder and IncResNet block and the 1D convolution transposed layer of the decoder was followed by batch normalization, Leaky ReLU with slope 0.2, and Inception-Res block.

The design of the Inception-Res block for the student model was kept the same as that of the teacher model. The convention of placing attention blocks and MC dropouts was kept the same as described in Section 4.3.5 and Section 4.3.6, respectively.

### 4.4. Experimental and Evaluation Details

All the experimental hyperparameters were fixed empirically; each of the two datasets was split into the ratio 80:20; train and test. The model was trained for 100 epochs, where we used the smooth *L*1 loss function as given in Equation (7), followed by applying the Adam optimizer. We selected a separate learning rate (LR) for each dataset for proper convergence of the loss function. The reason for using different learning rates lies in the nature of the DL model, which is task- and data-specific. For the PPG-DaLiA dataset, the learning rate is:

(23) $$LR=\left\{\begin{array}{cc} 1\;\times \;10^{-2}, & EPOCHS<=20\\ 1\;\times \;10^{-4}, & \mathrm{otherwise}. \end{array}\right.$$

For the IR dataset, the learning rate is:

(24) $$LR=\left\{\begin{array}{cc} 1\;\times \;10^{-2}, & EPOCHS<=20\\ 1\;\times \;10^{-3}, & \mathrm{otherwise}. \end{array}\right.$$

The model takes the intermediate respiration signal as the input vector. The input vector shapes for the PPG-DaLiA and the IR datasets are (814,128,3) and (722,128,3), respectively. The batch size was kept as 128. The attention blocks and MC dropouts were embedded into the architecture and trained based on the same specifications. For KD, the loss term is dependent on the regularization factor α. Hence, it is important to choose an optimal value of α such that both loss terms in Equation (22) contribute in the same order and result in proper feature distillation.

After careful examination, we fixed the value of α to 0.94. Other parameter specifications for the student model training were kept the same as the teacher model. Development was carried out in Tensorflow on a workstation housing an Nvidia GTX1080Ti 11 GB GPU.

The mean absolute error (MAE) and root mean square error (RMSE) were calculated between the average RR obtained from the IncResNet block and the ground truth RR. To compute the instantaneous RR, we obtain the individual breathing cycles using the output respiration signal. The error estimates for the same were calculated using the ground truth and the computed values. We evaluated our model against the previously developed DL/ML-based and basic signal processing algorithms. We also evaluated the performance improvement in the model by embedding the attention blocks and MC dropouts based on the MAE and RMSE. In the next section, we also demonstrate the model’s parameter count and inference time reduction.

## 5. Results and Discussion

### 5.1. Comparison with Previously Developed Techniques

Recently, DL models have consistently outperformed the traditional signal processing methods and ML algorithms. However, it is essential to analyze the increase in parameter counts and inference time along with the error scores to understand the practical difficulties in deploying a DL model.

We compared the proposed attention-mechanism-based multitasking network (ATTMRNet) with previously developed works. The metrics used for this evaluation were MAE, RMSE, parameter count (PC), and inference time taken (IT) per window. To this end, Table 2 consolidates the comparative results between the output from our model and the same from other methods (RRint: R–R-interval-based respiration algorithm, RPA: R peak amplitude-based respiration algorithm, ADR: accelerometer-based respiration algorithm, SF: smart fusion algorithm, LREG: linear regression algorithm, RN: RespNet, CNN: convolutional neural network, AVG RR: average RR, INST RR: instantaneous RR).

It is evident from Table 2 that the proposed model showed less error and time while using optimal parameters than the previously developed DL-based algorithms on both datasets. On the PPG-DaLiA dataset, the proposed model outperformed the basic approaches such as RRint, RPA, and ADR in terms of error scores. In comparison to the ML-based model, the proposed model showed better accuracy than SF and LREG. However, the IT in our model was significantly higher than LREG. The IT taken by our model was less than SF. When compared with DL-based approaches such as CNN and RN, our model showed less error for average and instantaneous RR. The PC used in our model was higher than CNN, while it was less than the same in RN. The IT for our model was also higher than CNN, and, on the other hand, it consumed similar IT to RN.

The model’s efficacy on high dynamic ambulatory activity can be seen when applying it to the IR dataset. Due to motion artifacts, the traditional methods have significant errors. The DL-based methods have significantly lower errors than the classical methods. Our proposed model showed the least error for average and instantaneous RR among all methods. The IT and PC taken for this dataset were similar to what we observed with the PPG-DaLiA dataset when compared with the other models.

Upon comparison with MRNet, the ATTMRNet showed a negligible difference in the average RR error for the PPG-DaLiA dataset. However, for the instantaneous RR, ATTMRNet showed lower error than MRNet.

ATTMRNet showed a lower error for the IR dataset for both average and instantaneous RR than MRNet. This reduction in error scores can be attributed to the usage of attention blocks, which are further elucidated in Section 5.2. The improvement in the error scores is achieved without any increment in IT but with a slight increase in PC.

Overall, considering the trade-off between the accuracy, PC, and IT, ATTMRNet performed effectively on both datasets in terms of error scores, but a significant reduction in PC and IT is still required.

### 5.2. Utility of Attention Block

The previous section shows that the proposed model gives lower errors than other methods. While this aspect of our model can be attributed to the usage of attention blocks, it is important to understand the effect of attention blocks on the output, as attention blocks make a DL model relatively interpretable.

Attention blocks suppress the irrelevant features while highlighting the salient features of the input. This helps the model to focus only on the portion relevant to the specific task, thereby improving the performance.

**Figure 3:**
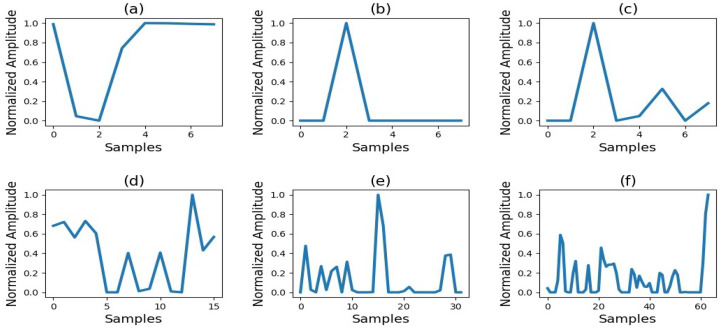
Step-by-step evolution of bottleneck feature maps towards the final respiration signal with the help of attention blocks’ output. Each subplot shows the feature map output of (**a**): bottleneck layer feature map; (**b**): first attention block feature map; (**c**): second attention block feature map; (**d**): third attention block feature map; (**e**): fourth attention block feature map; (**f**): fifth attention block feature map. The features are automatically suppressed or highlighted according to the weights generated by the attention block. The y-axis of each subplot represents the normalized amplitude of the feature maps. The x-axis of each subplot represents the number of samples in each feature map, whereas the last sample represents the feature map size.

The interpretability of the attention block can be understood from Figure 3, which shows the step-by-step evolution of the bottleneck layer feature maps towards a final estimate while suppressing the irrelevant features. Figure 3a represents the bottleneck layer features; these features are passed to the decoder for upsampling via the attention block. As per Figure 3, the sample size of the feature maps is increasing, while the irrelevant features are becoming suppressed to zero. The step-by-step process shown in Figure 3 leads to the feature map of the final attention block (Figure 3f), which resembles the respiratory waveform. Feeding these features as the input to the last stage of the decoder will generate the final output respiration signal.

In order to understand the impact of the attention block on the final respiration signal output, it is important to monitor the breathing peaks of the output with respect to the same in the reference respiration signal. However, the peak locations are discrete values and are different in length for the two signals; therefore, the direct calculation of MAE between the peak location of the two signals is difficult.

**Figure 4:**
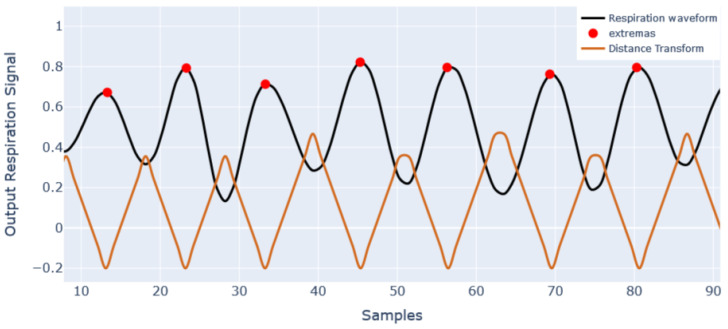
Random snippet of a final estimated breathing cycle andits distance transform.

Thus, to compare the peak locations of two waveforms, we applied the distance transform (DT) [46], which converts the discrete peaks into a continuous waveform, such that the location of peaks coincides with the minima of the distance transform, as given in Figure 4.

**Figure 5:**
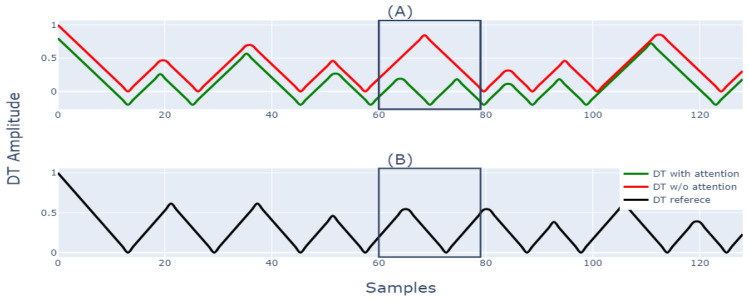
(**A**) shows the DT of respiration signal output with and without attention block; (**B**) shows the DT of reference respiration signal.

In order to understand the effect of the attention block, we performed a qualitative analysis between the DT of respiration signal obtained with and without the attention block against the DT of the reference respiration signal. The DT of all the signals is shown in Figure 5.

As the minima of DT represent the peak locations, the reference respiration signal has nine minima, i.e., nine peak locations. The DT with attention also has nine peaks, and its morphology is somewhat similar to DT of the reference respiration signal. However, the DT without attention missed one breathing peak, as shown in Figure 5 by a rectangular region. In the rectangular region, while both DT with attention and DT of reference signal attained a minimum, DT without attention did not attain a minimum. Performing the same qualitative analysis of other windows, it can be said that the respiration signal output with attention block closely matches the breathing peak location and morphology of the reference respiration signal compared to the respiration signal output without attention.

Additionally, the MAE between the DT of the two signals can be easily calculated. The smaller error with respect to the DT of the reference respiration signal shows that the respiration peak patterns of the two signals show a close resemblance to each other, resulting in a smaller error in instantaneous RR prediction. The MAE between the DT of the output waveform with attention block and DT of reference respiration signal is 0.16. On the other hand, the MAE between the DT of the output waveform without attention and the DT of reference respiration signal is 0.25; these error scores give a brief idea about the efficacy of the model with attention block in prediction. The detailed quantitative analysis of the efficacy of the model with attention block (ATTMRNet) against the DL models developed without attention block was already discussed in Section 5.1, which proved that the addition of attention blocks significantly reduced the error in the final prediction for both datasets. The overall discussion proves the effectiveness of attention blocks in reducing the error and providing insights into the model’s performance.

### 5.3. Evaluation of Predictions Based on Uncertainty

The application of attention blocks reduced the error scores, but for the application, during ambulatory settings, we are required to calculate the uncertainty in the prediction so that the less reliable predictions can be removed. The proposed model uses MC dropouts for uncertainty estimation. The MC dropouts are activated during inference, and the model needs to run multiple times to obtain the uncertainty associated with the final output, as described in Section 4.2.3. However, running the model multiple times increases the model’s IT. This makes selecting the optimal number of inference samples (*N*) crucial. The value of (*N*) should be selected so that the model takes a smaller IT and rejects the optimal number of uncertain windows from the predicted outputs. Unlike in Section 5.1, the IT here is considered for the entire test set. The results for the calculation of *N* for both datasets are consolidated in Table 3.

For N=5, the model shows relatively higher error for both average and instantaneous RR on both datasets. Therefore, N=5 cannot be considered for uncertainty estimation even though its IT is minimum. The window rejection rate for N=10,15,20 is almost the same for both datasets.

**Figure 6:**
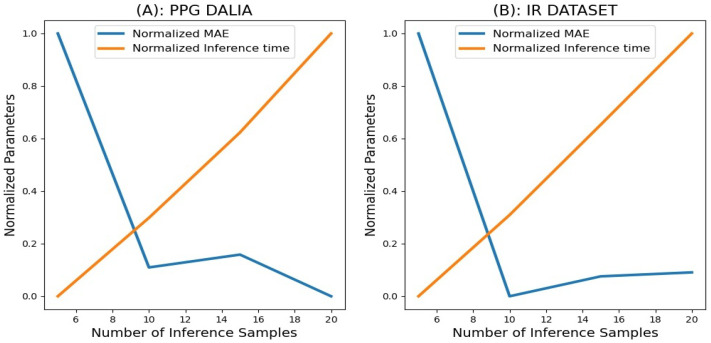
Number of inference sample vs, error and time. (**A**): Plot for PPG-DaLiA dataset; (**B**): plot for IR dataset.

Taking the insights from Figure 6, it is clear that the IT is very large for N=15,20. Therefore, N=15,20 cannot be considered as optimal choices for inference samples. It is evident from Figure 6 and Table 3 that N=10 consumed the optimal IT, while showing the error scores comparable to N=15,20 on both datasets. Hence, considering the trade-off between error, inference time, and percentage rejection of windows, N=10 proves to be a suitable choice to produce optimal results.

**Figure 7:**
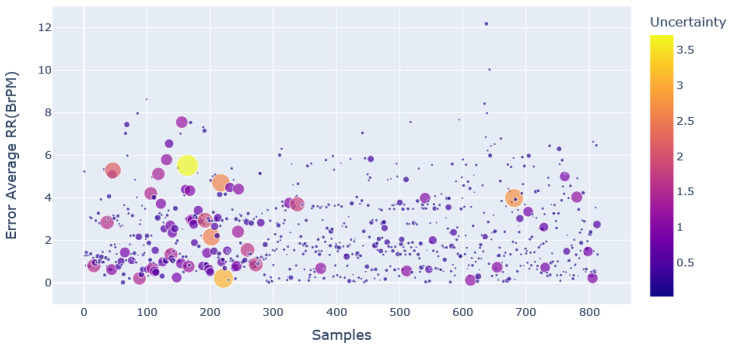
Scatter plot of uncertainty distribution with respect to error in average RR prediction.

**Figure 8:**
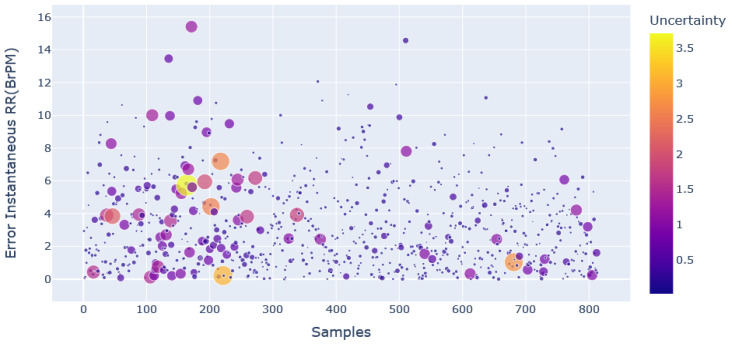
Scatter plot of uncertainty distribution with respect to error in instantaneous RR prediction.

The relations between the uncertainty estimate and the error that occurred in the prediction of average and instantaneous RR are shown in Figure 7 and Figure 8, respectively.

These two figures depict the uncertainty distribution that occurred in predicting average RR and instantaneous RR. The markers with a larger size show that the model has a higher uncertainty for that particular prediction.

These predictions should be neglected to enhance the model’s accuracy, and a scatter plot makes the process easier. It is evident from Figure 7 and Figure 8 that even though the error is low for a particular prediction, the model may not be confident about that prediction.

Some predictions also showed higher uncertainty for the higher error values. This implies that there is no direct relation between uncertainty estimate and errors.

The benefit of rejecting the windows based on uncertainty during inference is that the error calculation requires the ground truth data, while uncertainty estimation does not require any ground truth data. The criteria for rejecting windows based on uncertainty reduces the error significantly. The results of the MC dropout approach in uncertainty estimation, and thereby reducing the error, are shown in Table 4.

As given in Table 4, the error with MC dropout reduced significantly for both the PPG-DaLiA and the IR datasets. For the PPG-DaLiA dataset, the MAE and the RMSE for the average RR were reduced by 5.5% and 4.05%, respectively. On the other hand, the MAE and the RMSE for instantaneous RR were reduced by 5.06% and 0.7%, respectively. For the IR dataset, the MAE and the RMSE for the average RR were reduced by 10.48% and 8.73%, respectively. On the other hand, the MAE and the RMSE for instantaneous RR were reduced by 12.64% and 5.88%, respectively.

Among the two datasets, the significant reduction in errors for the IR dataset demonstrates the efficacy of the proposed model embedded with uncertainty estimation, thereby enhancing the accuracy for high-dynamic ambulatory data. The lower error scores for both the datasets are achieved by rejecting 3.80% of the total windows for the PPG-DaLiA dataset and 3.60% of the total windows for the IR dataset.

For the PPG-DaLiA dataset, out of all rejected uncertain windows,61.30% possessed the higher error for average RR, and 67.74% windows possessed a higher error for instantaneous RR. On the other hand, for the IR dataset, 46.15% uncertain windows possessed higher error for average RR and 65.38% windows possessed higher error for instantaneous RR. Here, error scores greater than 2 BrPM are considered as higher error values for both datasets. This proves that uncertainty estimation captures a considerable percentage of windows with high uncertainty and high error.

This deduction corroborates the usage of uncertainty estimation to enhance the accuracy of the final output. Overall, the performance of the model is improved by MC-dropout-based uncertainty estimation, which is essential for a model to be deployed in real-time settings.

### 5.4. Application of Knowledge Distillation

The previous section showed that the application of MC dropouts reduces prediction errors by removing predictions with higher uncertainty. However, the proper functioning of the model in real-time scenarios requires a reduction in the model’s parameter count and inference time. The application of KD helps in achieving the same.

A smaller-sized student model was selected to replicate the teacher model’s performance with a lower parameter count. Our base teacher model consisted of five levels of encoder and decoder ($n_{ed}=5$), as described in Section 4.3. Therefore, the student model can be designed by considering $n_{ed}=1,2,3,4$. However, as the size of the model decreases, the model tends to become inaccurate due to the reduction in parameter count, as described in [39].

In general, a student model is supposed to have a higher error in prediction compared to the teacher model. It is observed from studies such as [39,47] that while the student model is less accurate than the teacher model, the deviation in the accuracy of the student model is not too large. Therefore, a student model should be designed so that it shows a smaller increment in error with reference to the teacher model. To this end, we designed four student models with different levels of encoder and decoder, and the model showing the smallest increment percentage in the error with respect to the teacher model was selected for KD.

The results of this experiment are shown in Table 5. It is evident from Table 5 that $n_{ed}=3,4$ showed the lowest and almost the same increment in the error for both datasets. However, among $n_{ed}=3$ and $n_{ed}=4$, the former showed the lower PC. Therefore, $n_{ed}=3$ becomes the most suitable choice for the student model design for KD. The design specification of the student model is already described in Section 4.3.7.

Distilling the feature maps to the student model by teacher model helps the student model in imitating the teacher model in terms of accuracy with reduced PC and IT, as shown in Table 6.

The student model with a lower PC shows higher error than an original teacher trained without feature distillation. The proposed model with KD shows improvement in accuracy while operating at lower IT and PC for both datasets [22].

Here, also, the IT is calculated for the entire test set, unlike in Section 5.1. Apart from improvement in the error scores, the PC reduced by 49.5% in both the datasets. The reduction in IT was by 36.89% and 39.29% for the PPG-DaLiA and IR datasets, respectively. The MAE and RMSE after applying KD was reduced significantly compared to the larger teacher model. For the PPG-DaLiA dataset, the improvement in error scores of instantaneous RR is more significant than the improvement in average RR. For the IR dataset, improvement in error scores for both average and instantaneous RR is significant. Overall, the application of KD allows the reduction in parameter count and inference time while improving the accuracy.

### 5.5. Summary of Results and Discussion

From the overall analysis and discussion, the following inference can be drawn:

1. The proposed ATTMRNet performed significantly better than traditional methods and other ML/DL-based techniques in terms of error scores, as shown in Table 2. In comparison to the state-of-the-art MRNet [14], the ATTMRNet had lower error. This was demonstrated by a **0.4%** reduction in error for the average RR for PPG-DaLiA and **5.31%** for the IR dataset. For instantaneous RR, the error reduction was **7.64%** and **4%** for the PPG-DaLiA and IR datasets, respectively.
2. The addition of attention blocks helps to gain the interpretability of the model, which can be seen with the help of feature maps in Figure 3. The impact of attention block on the final output was shown by qualitative analysis in Figure 5.
3. Using MC dropouts for uncertainty estimation reduced the error significantly by rejecting **3.8%** of uncertain windows for PPG-DaLiA and **3.6%** for the IR dataset.
4. The application of KD reduced the model’s parameter count by **49.5%**. Consequently, a reduction in inference time by **36.89%** was observed for the PPG-DaLiA and **39.39%** for the IR dataset. Additionally, the application of KD also improved the final estimates’ accuracy when compared with the teacher model.

## 6. Conclusions

In this work, we introduced a multifunctional network to estimate average and instantaneous respiration rate with high accuracy, especially during ambulatory activities. The network is embedded with attention blocks, which helps in two ways. Firstly, it makes the model interpretable, and, secondly, it is also instrumental in reducing noise from the features caused by sudden movements. Pruning of irrelevant features reduced the error in the final prediction when compared with previously developed methods. We further scrutinized the noise that crept in during prediction by removing predictions with high uncertainty obtained by applying MC dropouts into the same network. This aspect enabled the model to reject highly uncertain windows, thereby increasing the accuracy. Lastly, we added knowledge distillation to reduce the computation time and memory occurring due to the requirement of running the model multiple times to estimate uncertainty. Overall, adding the components mentioned above makes the model robust to high dynamic activities and makes it optimal for deploying on wearable edge devices.

In our future work, we intend to deploy the model on the Raspberry Pi 4 to test its utility on edge devices. Additionally, we would like to explore the concept of evidential deep learning for uncertainty estimation to reduce the number of inference samples from the current value of ten to one. 

## Figures and Tables

**Table 1:** Details of speed and slopes used in each stage of Bruce protocol.

STAGE	TIME (Min)	SPEED (km/h)	SLOPE (%)
1	0	2.7	10
2	3	3	12
3	6	5.4	14
4	9	6.7	16
5	12	8	18
6	15	8.8	20
7	18	9.6	22
8	21	10.4	24
9	24	11.2	26
10	27	12	28

**Table 2:** Comparison between previously developed works and proposed ATTMRNet.

DATASET	METHOD	AVG RR		INST RR		PC (M)	IT (s)
**PPG-DaLiA**		**MAE**	**RMSE**	**MAE**	**RMSE**		
	RRint	3.26	4.13	3.54	4.5	NA	$1.1\;\times \;10^{-3}$
	Rpeak	3.46	4.37	3.82	4.85	NA	$1.1\;\times \;10^{-3}$
	ADR	3.15	3.96	3.5	4.35	NA	$1.1\;\times \;10^{-3}$
	SF	2.94	3.88	NA	NA	NA	0.48
	LREG	3.46	4.7	NA	NA	NA	$1.5\;\times \;10^{-4}$
	CNN	2.8	3.56	NA	NA	0.45	0.01
	RN	3.22	4.02	3.34	4.18	39.14	0.103
	MRNet	2.37	2.97	3.42	4.31	23.06	0.11
	**ATTMRNet**	**2.36**	**2.97**	**3.16**	**3.97**	**24.82**	**0.11**
**IR DATASET**	RRint	4.35	5.4	4.18	5.24	NA	$1.1\;\times \;10^{-3}$
	Rpeak	3.92	4.99	3.86	4.94	NA	$1.1\;\times \;10^{-3}$
	ADR	4.21	5.14	4.5	5.6	NA	$1.1\;\times \;10^{-3}$
	SF	4.07	5.37	NA	NA	NA	0.15
	LREG	4.25	5.55	NA	NA	NA	$1.7\;\times \;10^{-4}$
	CNN	3.17	3.99	NA	NA	0.45	0.01
	RN	3.57	4.46	3.85	4.5	39.14	0.12
	MRNet	2.82	3.57	3.54	4.39	23.06	0.12
	**ATTMRNet**	**2.67**	**3.55**	**3.4**	**4.25**	**24.82**	**0.11**

**Table 3:** Description of trade-off between the number of inference samples, number of uncertain windows rejected, and the inference time consumed.

DATASET	INFERENCE SAMPLES	AVG RR		INST RR		% WINDOWS REJECTED	IT (s)
**PPG-DaLiA**		**MAE**	**RMSE**	**MAE**	**RMSE**		
	5	2.27	2.88	3.73	4.8	3%	8
	10	2.23	2.85	3	3.94	3.80%	13.9
	15	2.23	2.84	3.04	3.88	4%	20.32
	20	2.23	2.85	2.91	3.8	4%	27.76
**IR dataset**	5	2.45	3.27	3.63	4.68	5.12%	7.16
	10	2.39	3.24	2.97	4	3.60%	12.08
	15	2.48	3.27	3.02	3.95	3.30%	17.54
	20	2.48	3.27	3.03	3.94	2.90%	23.06

**Table 4:** Description of error reduction due to application of Monte Carlo dropout for both datasets.

DATASET	METHOD	AVG RR		INST RR	
		**MAE**	**RMSE**	**MAE**	**RMSE**
**PPG-DaLiA**	Without MC dropout	2.36	2.97	3.16	3.97
	**With MC dropout**	**2.23**	**2.85**	**3.01**	**3.89**
**IR dataset**	Without MC dropout	2.67	3.55	3.4	4.25
	**With MC dropout**	**2.39**	**3.24**	**2.97**	**4**

**Table 5:** Comparison among the student model designed with various level of encoder and decoder.

Number of Encoder and Decoder Levels ($n_{\mathrm{ed}}$)	Percentage Increment in Error for PPG-DaLiA Dataset	Percentage Increment in Error for IR Dataset	PC (M)
1	16.45%	8.07%	10.91
2	4.76%	7.3%	11.09
**3**	**2.16%**	**2.69%**	**12.53**
4	2.45%	2.69%	16.97

**Table 6:** Comparison between teacher model, student model, and proposed model with KD.

DATASET	METHOD	AVG RR		INTSANT RR		PC(M)	IT (s)
		**MAE**	**RMSE**	**MAE**	**RMSE**		
**PPG-DaLiA**	Teacher model	2.31	2.91	3.14	4.01	24.8	15.15
	Student model	2.36	2.95	3.34	4.13	12.53	9.68
	**KD model**	**2.22**	**2.87**	**2.81**	**3.66**	**12.53**	**9.56**
**IR dataset**	Teacher model	2.6	3.47	3.09	4.18	24.8	13.41
	Student model	2.67	3.5	3.23	4.23	12.53	8.32
	**KD model**	**2.38**	**3.17**	**2.81**	**3.73**	**12.53**	**8.14**

## Data Availability

The relevant code and datasets used in this study are available at our GitHub Repository. https://github.com/Acrophase/ATTMRNet.git.

## Abbreviations

The following abbreviations are commonly used in this manuscript:

RR	Respiration rate
ECG	Electrocardiogram
PPG	Photoplethesmography
RRint	R–R-interval-based respiration signal
Rpeak	R-peak-based respiration signal
ADR	Accelerometer-derived respiration
DL	Deep learning
ML	Machine learning
LR	Learning rate
IT	Inference time
PC	Parameter count
MC	Monte Carlo
KD	Knowledge distillation
